# Thrombotic and hemorrhagic events in critically ill COVID-19 patients: a French monocenter retrospective study

**DOI:** 10.1186/s13054-020-03025-y

**Published:** 2020-06-02

**Authors:** Megan Fraissé, Elsa Logre, Olivier Pajot, Hervé Mentec, Gaëtan Plantefève, Damien Contou

**Affiliations:** Service de réanimation polyvalente, Centre Hospitalier Victor Dupouy, 69, rue du Lieutenant-Colonel Prud’hon, 95100 Argenteuil, France

**Keywords:** SARS-CoV-2, COVID-19, Thrombosis, Pulmonary embolism, D-dimers, ARDS, Intensive care

## Introduction

Patients admitted to the intensive care unit (ICU) for coronavirus disease 2019 (COVID-19), following an infection by the severe acute respiratory syndrome coronavirus 2 (SARS-CoV-2), seem to have an increased risk of thrombotic events (TE), both arterial and venous [1–3]. Even in the absence of high level of evidence, some centers have consequently modified their pharmacological thromboprophylaxis strategy toward intermediate or full-dose (therapeutic) preemptive anticoagulation (rather than prophylactic dosing) for routine care of COVID-19 patients [2, 4], exposing them to a higher risk of hemorrhagic events (HE).

Our aim was to assess the rates and to describe each TE and HE occurring in critically ill COVID-19 patients admitted to our ICU.

## Methods

We retrospectively analyzed data from patients admitted to our 41-bed COVID-19 ICU between March 6 and April 22, 2020. All patients received usual (prophylactic) or full-dose (therapeutic) anticoagulation according to their risk factors for thrombosis [4]. All TE (venous or arterial) diagnosed during ICU stay were collected, except central venous catheter related thrombosis and hemodialysis filter thrombosis. TE were investigated only in case of clinical suspicion, and not in a routine manner. All significant (interruption of anticoagulation and red blood cell transfusion, surgical intervention or death) HE were collected.

## Results

**Fig. 1:**
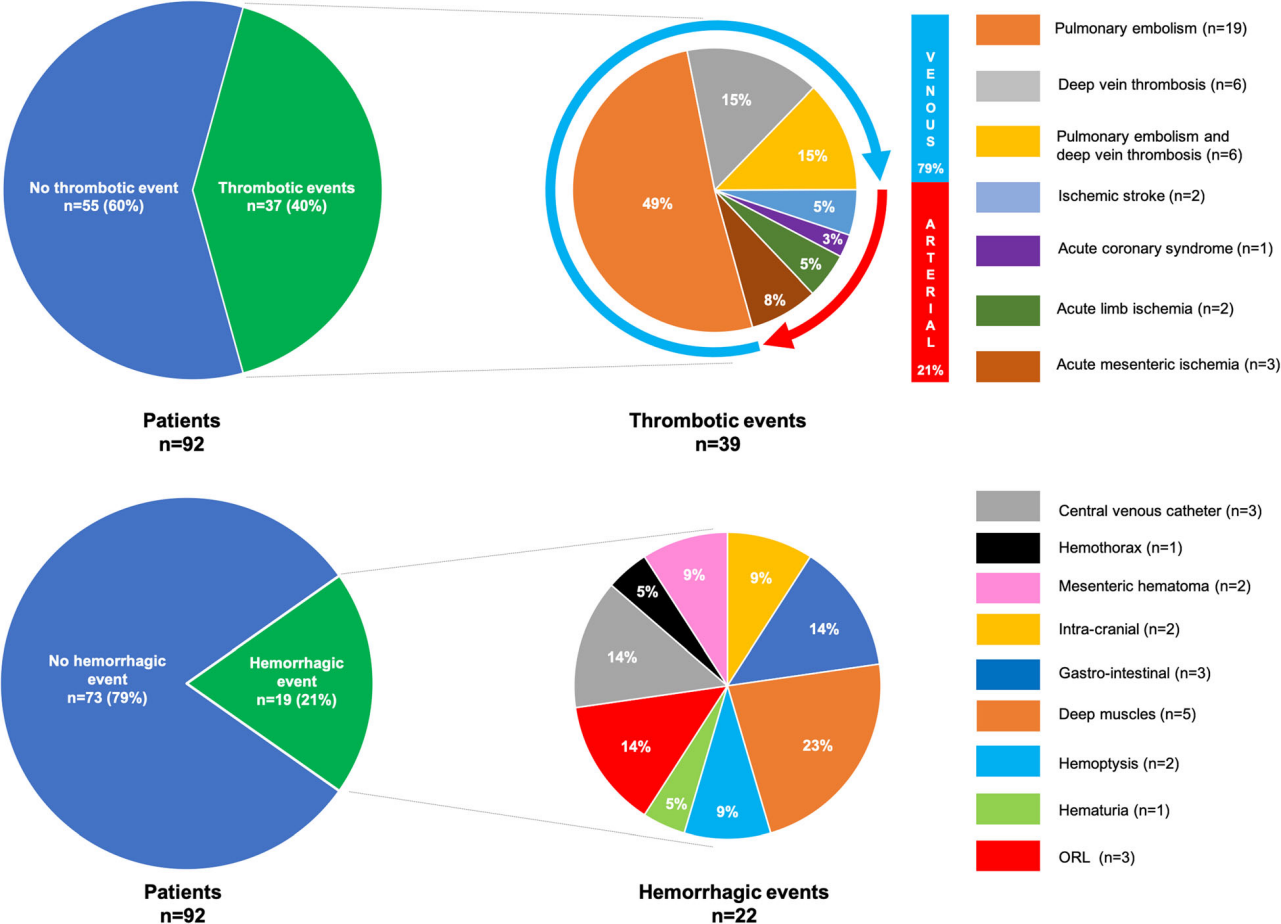
Description of thrombotic and hemorrhagic events among 92 critically ill COVID-19 patients

During the study period, 92 patients were admitted to our ICU for acute respiratory failure related to SARS-CoV-2 pneumonia. Main demographic and clinical characteristics are detailed in the Table 1. At the time of analysis (May 6), 37 of them (40%) experienced a total of 39 TE including 31 venous (79%) and 8 arterial (21%) thrombosis, and 19 of them (21%) experienced a total of 22 HE during their ICU stay. Distributions of each TE and HE are detailed in the Fig. 1.

**Table 1:** Characteristics of 92 critically ill COVID-19 patients who developed (*n* = 37, 40%) or not (*n* = 55, 60%) a thrombotic event during ICU stay (*at the time of analysis (May 6, 2020); data for 91 patients as one patient was lost to follow-up)

	All patients, ***n*** = 92	Patients without thrombotic events, ***n*** = 55 (60%)	Patients with thrombotic events, ***n*** = 37 (40%)	***p***
Age (years)	61 [55–70]	61 [55–69]	62 [54–71]	0.89
Male, *n* (%)	73 (79)	44 (80)	29 (78)	0.55
Body mass index (kg/m^2^)	30 [26–35]	32 [25–36]	29 [27–34]	0.34
Baseline SOFA	4 [3–7]	4 [2–7]	4 [3–8]	0.6
Baseline SAPS II	31 [21–40]	31 [20–39]	31 [22–44]	0.8
**Main comorbidities,*n*(%)**				
Hypertension	59 (64)	36 (65)	23 (62)	0.75
Diabetes mellitus	35 (38)	22 (40)	13 (35)	0.75
Cardio-vascular diseases	9 (10)	8 (14)	1 (3)	0.48
Atrial fibrillation	3 (3)	1 (2)	2 (5)	0.06
Cerebro-vascular diseases	8 (9)	5 (9)	3 (8)	0.34
Venous thrombo-embolism	5 (5)	3 (5)	2 (5)	0.89
Chronic respiratory diseases	18 (20)	11 (20)	7 (19)	0.99
Chronic renal failure	7 (8)	7 (13)	0	0.03
**Laboratory measurements at ICU admission**				
Fibrinogen (g/L)	7.8 [6.1–8.8]	7.5 [6.0–8.6]	7.9 [6.3–9]	0.45
D-dimers (μg/mL)	2.4 [1.7–7.9]	2.2 [1.2–5.9]	4.4 [1.8–2]	0.03
Prothrombin time (%)	86 [76–96]	92 [80–100]	79 [68–90]	< 0.001
Platelets (G/L)	227 [182–307]	213 [170–302]	235 [198–340]	0.17
**Thromboprophylaxis strategy**				
Usual (prophylactic) anticoagulation	43 (47)	26 (47)	17 (46)	0.90
Full-dose (therapeutic) anticoagulation	49 (53)	29 (53)	20 (54)	0.90
**Outcome in ICU,*n*(%)**				
Invasive mechanical ventilation	82 (89)	46 (84)	36 (97)	0.04
Prone positioning	55 (60)	32 (58)	23 (62)	0.70
Vasopressor support	57 (62)	31 (56)	26 (70)	0.18
Renal replacement therapy	22 (24)	11 (20)	11 (30)	0.28
ICU mortality*	38 (41)	20 (36)	18 (49)	0.24
Still hospitalized in ICU*	25 (27)	13 (24)	12 (32)	0.35
Discharged to the wards*	28 (30)	21 (38)	7 (19)	0.05

Continuous variables were reported as median [Interquartile range] (IQR) and compared between groups using the Mann-Whitney test. Categorical variables were reported as numbers and percentages and compared using *χ*^2^ test or Fisher’s exact test, as appropriate. A *p* value < 0.05 was considered significant

Median days between ICU admission and TE and between first COVID-19 symptoms and TE were 9 [3–21] and 17 [12–28], respectively. There was no difference regarding clinical characteristics between patients with or without a TE, except from D-dimer levels and prothrombin time (Table 1). Among patients with an HE, 16 (*n* = 16/19, 84%) received full-dose anticoagulation, including 8 (50%) without a confirmed TE. All HE required interruption of anticoagulation, 3 (14%) were fatal 1 (2 intra-cranial, 1 deep muscle), 14 (64%) required red blood cells transfusion, and 5 (24%) required bronchoscopy or gastroscopy. Only one of the 15 (7%) anti-Xa activity performed the day of HE was overdosed.

## Discussion

We herein report on a 40% rate of TE in critically ill patients with COVID-19, mostly venous TE. This rate is in accordance with previous series [1, 2]. However, it is lower than the 69% rate reported by Llitjos et al. who performed a systematic screening with Doppler ultrasound [5]. As previously reported [2], PE was the leading cause of thrombosis in our cohort. As there is a growing interest in chest CT scan as a diagnostic tool for SARS-CoV-2 pneumonia [6], performing a systematic pulmonary angiography may constitute a relevant strategy.

Noteworthy, we report on a 21% rate of significant HE, most of them occurring in patients with full-dose anticoagulation. As half of these patients were treated with full-dose preemptive anticoagulation without a confirmed TE, we must be cautious about our thrombo-prophylaxis strategy with daily reassessment of its indication. Moreover, it is interesting to note that full-dose anticoagulation did not prevent some patients from developing a TE.

The retrospective monocenter design of our study implies numerous limitations. Nevertheless, we reported on a high rate of TE and HE in ICU COVID-19 patients highlighting the necessity to adapt our thrombo-prophylaxis strategy as well as our TE screening strategy.

## Data Availability

The dataset used and analyzed for the current study is available from the corresponding author on reasonable request.

## Abbreviations

SOFA: Sequential Organ Failure Assessment
SAPSII: Simplified Acute Physiology Score II
